# Neural Adaptation Provides Evidence for Categorical Differences in Processing of Faces and Chinese Characters: An ERP Study of the N170

**DOI:** 10.1371/journal.pone.0041103

**Published:** 2012-07-24

**Authors:** Shimin Fu, Chunliang Feng, Shichun Guo, Yuejia Luo, Raja Parasuraman

**Affiliations:** 1 Department of Psychology, Tsinghua University, Beijing, China; 2 State Key Laboratory of Cognitive Neuroscience and Learning, Beijing Normal University, Beijing, China; 3 Department of Psychology, George Mason University, Fairfax, Virginia, United States of America; University of British Columbia, Canada

## Abstract

Whether face perception involves domain-specific or domain-general processing is an extensively debated issue. Relative to non-face objects and alphabetical scripts, Chinese characters provide a good contrast to faces because of their structural configuration, requirement for high level of visual expertise to literate Chinese people, and unique appearance and identity for each individual stimulus. To examine potential categorical differences in their neural processing, event-related potentials (ERPs) were recorded to blocked face and Chinese character stimuli. Fast adaptation method was applied to better control for the low-level stimulus difference between faces and Chinese characters. Participants were required to respond to the color of the outer frame in which these stimuli were presented, at either a fast (ISI 650 ms) or slow (ISI 1300 ms) rate, and with an orientation that was either the same or alternated between upright and inverted. Faces elicited a larger and later N170 relative to characters, but the N170 was more left-lateralized for characters relative to the faces. Adaptation-by-rate and adaptation-by-orientation effects were observed on the amplitude of N170, and both were more pronounced for faces relative to characters. Inverted stimuli elicited a later N170 relative to upright stimuli, without amplitude change, and this inversion effect was more pronounced for faces relative to characters. Moreover, faces elicited a larger and later P1 and a larger adaptation-by-rate effect on P1 relative to characters. The adaptation-by-orientation effect was illustrated by a larger P1 under the same relative to the alternated orientation condition. Therefore, evidence from the amplitude and the lateralization of N170, the stimulus inversion effect on N170 latency, and the neural adaptation between faces and Chinese characters on P1 and N170 components support the notion that the processing of faces and Chinese characters involve categorically different neural mechanisms.

## Introduction

Whether the human visual system processes faces in a special, domain-specific way has been an issue for debate and controversy. Behavioral studies have shown that the inversion effect is larger for faces relative to other objects [1]. Brain imaging studies have also shown that a specific region in the brain, the fusiform face area (FFA), is activated more by faces than by non-biological objects such as houses, chairs, or cars [2], supporting the domain-specific view [3]. Consistent with this view, single-unit recordings in macaques have found stronger response to faces relative to non-face objects in the same fMRI-identified face region [4], and event-related potential (ERP) studies have pointed to a face-specific component, N170 [5], [6]. However, the domain-specific view of face processing has been challenged by a domain-general view [7]. Studies with experts in classifying non-human objects such as cars and birds [8] and fingerprints [9] and with individuals trained to become experts at recognizing “greebles” (computer-generated non-face stimuli comparable to faces in their attributes; [10]), have suggested that visual expertise, rather than domain-specificity, is the key factor in this pattern results. Because humans have encountered thousands of faces repeatedly during their lifetime, they are “face experts.” According to this view, then, expertise rather than the category-specific nature of faces, is responsible for activation of FFA and for changes in the amplitude and latency of the electrophysiological counterpart, the N170 [11], [12]. Therefore, it is still controversial whether faces are a “special” category of visual objects and how visual expertise affects this processing.

Relative to other objects and alphabetical scripts, Chinese characters may provide a better set of contrasting stimuli for comparison with faces than other non-face objects or artificial stimuli like greebles [13], [14]. Firstly, for skilled Chinese readers, Chinese characters are similar in many ways to faces: they require considerable visual expertise, are unique in visual appearance and identity, have canonical upright orientation, and possess a squared visual configuration consisting of parts (strokes and radicals) relative to other objects (e.g., cars) and alphabetic scripts (e.g., English words). Chinese characters, just like faces, are also ubiquitous in daily life for Chinese people, and literate Chinese adults are experts who can read approximately the 3,000 most frequently used Chinese characters after years of learning. Thus it is likely that native literate Chinese people are experts for faces and Chinese characters, at a comparable or higher level relative to other visual objects, such as houses and cars. Moreover, relative to other visual objects, faces and Chinese characters are each characterized by many different category-specific exemplars. In addition, the linear (non-squared) configuration of English words makes it difficult to control for stimulus size and configuration when comparing them to faces. Therefore, contrasting Chinese characters and faces in Chinese speakers can enable a strong test of the domain-specific theory, given that the close match of the two object categories on other non-categorical features and low-level stimulus properties.

To further test the domain-specific versus domain-general views, the present study examined the N170, a negative-going ERP component with a peak latency of about 140–180 ms post-stimulus, for faces and Chinese characters. Previous research has revealed many known characteristics of the N170 elicited by face stimuli. The face N170 (1) is larger in amplitude than that for other objects, such as hands [6], cars, houses and chairs [15], or birds and furniture [16]. (2) It has a maximal amplitude at electrode sites over occipito-temporal scalp sites. (3) N170 is often (but not always) larger in the right than in the left hemisphere [17]. (4) It has a later peak latency for inverted relative to upright faces [18], [19]. (5) It is larger or of comparable amplitude for inverted relative to upright faces [20]. Finally, (6) N170 is thought to have neural generators in the lateral inferior occipital cortex and posterior fusiform gyrus [19], [21] close to FFA and middle/superior temporal areas (see [17], for a review). Recent studies with acquired prosopagnosia patients have pointed the neural generators for face-sensitive N170 to middle fusiform gyrus and superior temporal sulcus [22] or occipital face area (OFA, [23]).

In contrast to these well-established features of the face-related N170, the characteristics of the N170 for Chinese characters are less clear. Previous studies have shown a left-lateralized N170 for experts of both alphabetic scripts (e.g., English words and Japanese Kana [19], [24], [25], [26]) and non-alphabetic characters (e.g., Korean characters and Japanese Kanji, see [26], [27]). Moreover, the left-lateralized N170 for both syllabic and logographic Japanese scripts was observed for Japanese readers but not for English-only readers, pointing to a role for visual expertise in brain processing [26]. Less well known is the lateralization of processing Chinese characters. Domain-specificity in processing Chinese characters has been suggested by fMRI studies showing distinctive activation patterns for Chinese characters compared to alphabetical pinyin scripts [28], [29] and by meta-analysis of imaging studies showing an activation in inferior occipito-temporal cortex for reading Chinese characters only, in addition to common activations for reading Chinese characters, alphabetical (e.g., English) and Japanese scripts [30], [31], and bilateral activation was found in inferior occipito-temporal area for reading Chinese [31]. Consistent with this view that processing of Chinese characters may involve a category-specific form of processing, a larger N170 was observed for Chinese characters relative to English and Romans, with a more left-lateralized N170 of Chinese characters for bilinguals (English and Chinese) relative to English-only participants [32]. Therefore, the processing of Chinese characters may, like faces, involve “special” processing in the brain, although the hemispheric lateralization of N170 to such stimuli is still unclear.

The present study used an ERP-adaptation technique to test the domain-specific model of face and Chinese character processing. Neural adaptation, whether reflected in ERPs, fMRI, or other brain measures, refers to the phenomenon that neural responses to a stimulus category become smaller when the category is repeated (e.g., [33] for magnetoencephalographical evidence). It has been shown that adaptation of an original face for a period of 180 s can alter participants’ behavioral judgment of the gender, ethnicity, and expression of subsequently presented morphed faces [34]. Electrophysiological studies have also shown that adaptation of faces reduces the amplitude of N170 or its MEG counterpart, the M170 [35], [36], [37], [38], [39]. For example, the N170 in the right hemisphere was even smaller for identity adaptation, when a front-view face was adapted for approximately 3 seconds by a 30-degree-rotated face with the same relative to a different identity [40]. A similar N170 reduction effect for faces was observed with fast stimulus presentation rate (e.g., 200 ms ISI [41]). Therefore, first of all, adaptation is a robust visual phenomenon that provides critical information of the neural mechanisms of the categorical level at which different stimuli are processed. Specifically, if faces and Chinese characters involve categorically different neural mechanisms, then different ERP adaptation effects for faces and characters are expected. More importantly, physical differences are often a confound in studies directly comparing different stimuli (e.g., faces vs. cars or faces vs. words), because the early ERP components (such as P1, and N1 which overlaps with the N170 in face studies) are sensitive to low-level physical properties, such as spatial frequency, stimulus size, and luminance [5], [42]. Such confound of physical property difference is present in ERP studies comparing faces with words and other objects [25], [26]. In contrast, the comparison between the adaptation effects of faces and Chinese characters is not dependent on the physical difference between these two types of stimuli, because the effects of physical difference can be balanced within each category. Therefore, the use of adaptation paradigm in the present study should help to reveal categorical difference without being confounded by low-level physical differences between faces and Chinese characters.

The present study had the following aims. Firstly, we sought to characterize the response properties of the N170, especially the hemispheric distribution of N170 for faces and Chinese characters. Secondly, we addressed whether there is a categorical difference between faces and Chinese characters when these two stimuli are adapted under different presentation rates (fast vs. slow presentation rate, i.e., adaptation by rate) and under different stimulus sequence (same vs. alternating orientation, i.e., adaptation by orientation). We predicted that N170 for faces and Chinese characters should have different response properties and lateralization if they involve categorically “special” processing. We also predicted that the N170 for faces and Chinese characters should be reduced after adaptation by a fast presentation rate and by the same stimulus orientation. Moreover, these adaptation effects on N170 should indicate whether the processing of faces and Chinese characters involve distinctive neural mechanisms. Therefore, the present study used a design that should provide further ERP evidence relevant to the theoretical debate on the domain-specific or domain-general view of face perception, with better controls for low-level physical properties and comparable visual expertise between the two stimulus categories.

## Methods

### Participants

Eighteen healthy students (9 males) aged 18–27 years (mean age 21.9 years) participated in the experiment as paid volunteers. They were all native Chinese who had more than 10 years of education in speaking and reading Chinese characters. They were all right handed, had normal or corrected to normal vision, and had no neurological history. The experimental procedure and the recruitment of participants were approved by the Institutional Review Board (IRB) at Beijing Normal University. Informed written consents were obtained from all participants.

### Stimuli

**Figure 1:**
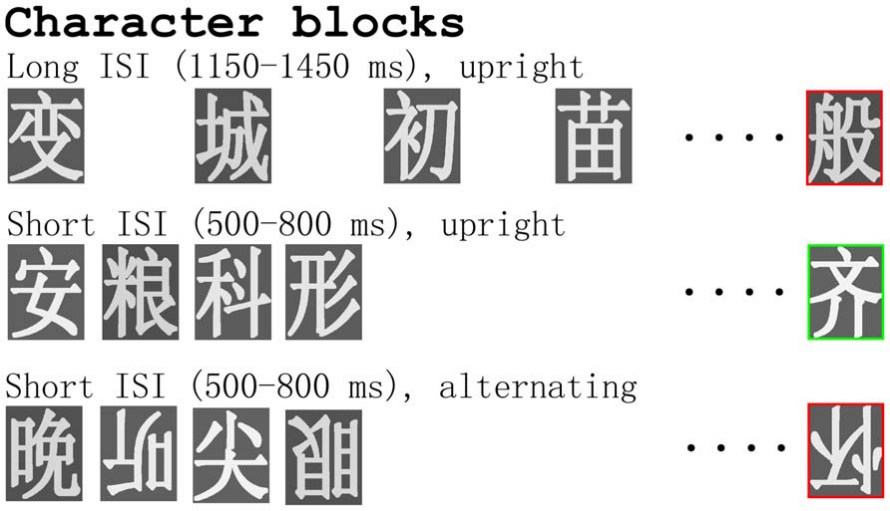
Illustration of the procedure of the present study. Only the Chinese characters are shown in this figure. Faces and Chinese characters were presented in separate blocks. Participants were instructed to respond to the targets (stimuli with a red or green rectangle frame, 11.76% of total trials) by pressing one key for a red and another key for a green rectangle. There were different types of stimulus blocks for faces and Chinese characters: upright blocks (the upper 2 rows) with long (1150–1450 ms, mean 1300 ms) or short (500–800 ms, mean 650 ms) ISI, inverted blocks (not shown in this figure) with long or short ISI, and alternated blocks (the lowest rows) with short ISI. The contrasts between the upper 2 rows were the “adaptation-by-rate” effects, and the contrasts between the lower 2 rows were the “adaptation-by-orientation” effects for upright stimuli, respectively. The same contrasts were also performed for inverted faces and characters (not shown in this figure).

Stimuli were 76 neutral faces and 76 Chinese characters. Face pictures were chosen from the Chinese Facial Affective Picture System (CFAPS, State Key Laboratory of Cognitive Neuroscience and Learning, Beijing Normal University)1, including 38 male faces and 38 female faces. All the faces had a neutral expression. Chinese characters were chosen from Modern Chinese Corpus of Center for Chinese Linguistics, Peking University (http://ccl.pku.edu.cn:8080/ccl_corpus/index.jsp?dir=xiandai). The frequency of each character was higher than 0.11/1000 in this Corpus, with a mean of 0.54/1000, and the stroke number of characters ranged between 6 and 13 (mean 9.0). There was no repetition among characters on their radicals and pronunciations (Figure 1). There were equal number of the left/right and up/down structured characters. Characters were printed in 200-point *Song* font, and bolded with 15 pixels. Stimulus size (faces or characters) was 4.53°×3.55°. The luminance and contrast grade between faces and characters were matched to a pre-selected face picture. A red or green frame (10 pixels in thickness) was added to16 face pictures and 16 character pictures. The number of red and green frames was equal for both faces and characters. Inverted stimuli were created by rotating the face and character pictures by 180°.

### Procedure

Faces and Chinese characters were presented at the center of a screen with a white background. Stimulus duration was 200 ms. The presentation of faces and Chinese characters was blocked, with 6 blocks each of faces and Chinese characters (see Figure 1). The 6 blocks of stimuli were: (a) 1 block of upright stimuli with long ISI, and 1 block of inverted stimuli with long ISI; (b) 1 block of upright stimuli with short ISI, and 1 block of inverted stimuli with short ISI; and (c) 2 blocks of stimuli with alternating orientation and short ISI. The short ISI was randomized between 500 and 800 ms (mean ISI 650 ms), and the long ISI was randomized between 1150 and 1450 ms (mean ISI 1300 ms). Participants performed all the face blocks first and then character blocks, or vice versa, in a counter-balanced order across participants. The sequence of 6 blocks within each category was randomized for each participant. Participants had a rest period of at least 1.5 minutes after each block. There were 136 trials in each block, with 120 non-target trials and 16 target trials. The non-targets consisted of 60 different pictures that were presented twice in each block. The targets consisted of 16 unrepeated pictures, for both the face and Chinese character blocks.

Participants were seated in a dimly-lighted and sound-attenuated room, with their eyes 100 cm away from the screen. They were instructed to fixate on the center of the screen and respond to the color of the frame for targets (either red or green with equal probability) as quickly and accurately as possible. Participants were required to discriminate the color of the frame and press “N” with right index finger for red frame or “B” with left index finger for green frame. The response hands to the two colors were balanced across participants. No responses were required to the stimuli without colored frame (i.e., non-targets). Participants were required to minimize eye blinks and eye movements. Short blocks of practice (10 trials each) were given to the participants until they were familiar with the task.

### EEG Recording

EEG was recorded from 64 scalp sites using Ag/AgCl electrodes mounted on a Quick-cap (Compumedics, Texas, USA), with the physical reference on the left mastoid. Vertical electro-oculographic (EOG) activity was recorded from electrodes above and below the left eye, and horizontal EOG was recorded from electrodes placed at the outer canthi of both eyes. Impedance was maintained below 5 kΩ throughout the whole recording session. The electroencephalogram (EEG) and EOG were amplified using a 0.05–100 Hz band-pass and continuously sampled at 500 Hz.

### Data Analysis

In the present study, faces and Chinese characters were presented in separate blocks, with upright and/or inverted orientation. Sensory adaptation effects of faces and Chinese characters were investigated by rate contrast and sequence contrast. For the rate contrast, all stimuli in each block were in the same category (either face or Chinese characters) and had the same orientation (either upright or inverted), but the presentation rate was long (mean 1300 ms) or short (mean 650 ms) in different blocks (Figure 1, upper 2 panels). For the sequence contrast, the presentation rate was kept the same (mean 650 ms), but the orientation of the stimuli (upright and inverted) could be the same or alternating in different blocks (Figure 1, lower 2 panels).

Electrophysiological data were analyzed by using Neuroscan 4.3.1 software (Compumedics, Texas, USA). The EEG analyzing window was between −200 to 800 ms, with the 200 ms pre-stimulus EEG served as baseline. EEG data were band-pass filtered with a range of 0.1–40 Hz. EEGs were first re-referenced to the algebraic average of LM/RM and then were re-referenced to the average of all the electrodes. Artifact rejection was performed for all the EEG channels and the rejection criteria was ±75 µV.

Only the ERPs of the non-target stimuli were analyzed. ERP analyses were separately performed for the contrasts between two presentation rates (short vs. long ISI) and between two sequences (same vs. different orientation). Two electrode pairs, one at the lower occipito-temporal area (PO7/PO8), and the other at the temporal areas (P7/P8), were selected for statistical analysis. Repeated measures of ANOVA were used for statistical analysis. This selection is based on the face literature showing largest N170 of faces at the occipito-temporal and temporal sites. The analysis of the P1 component was also based on these two temporal and occipito-temporal electrode pairs. For contrast between rates, the factors were Stimuli (faces vs. characters), ISI (long vs. short), Electrode (PO7/PO8 vs. P7/P8), Hemisphere (left vs. right), and Orientation (upright vs. inverted). For contrast between sequences, the ISI factor was replaced by the Sequence factor (same vs. alternating orientation).

## Results

### Behavioral Results

The false alarm rates were less than 0.1% for both faces and Chinese characters. The mean hit-rate for all the targets (stimuli with red or green outlines) was 93.1% ±4.3%. Reaction times were collapsed across the red and green targets. For the rate contrast, participants responded faster to targets under short relative to long SOA condition [530 vs. 573 ms, F (1, 17) = 87.635, p<.0005]. This rate effect was more pronounced for upright relative to inverted targets (53 vs. 33 ms), as suggested by a significant rate × orientation interaction [F (1, 17) = 5.798, p<.028]. No other main effects or interactions were significant. For the sequence contrast, no difference in RTs was found between the alternating and same orientation conditions [533 vs. 530 ms, F (1, 17) = 1.192, p<.290]. Participants responded faster to upright relative to inverted targets [528 vs. 535 ms, F (1, 17) = 6.141, p<.024]. This RT advantage to upright stimuli was more pronounced for faces relative to characters [17 vs. −3 ms], as suggested by a significant Stimuli × Orientation interaction [F (1, 17) = 9.154, p<.008].

### Electrophysiological Results

**Figure 2:**
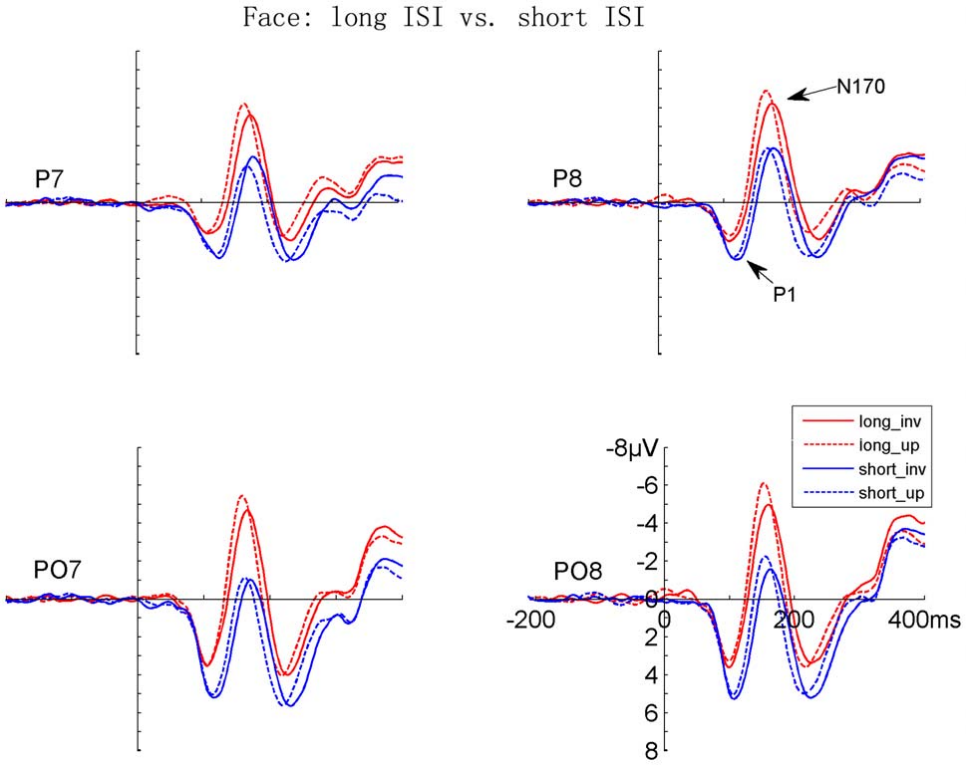
The grand-averaged ERPs elicited by upright and inverted faces under long and short ISI conditions. Data from the occipito-temporal (PO7/PO8) and temporal (P7/P8) sites are shown. The ERPs under long and short ISI conditions are denoted by red and blue lines, respectively. The ERPs for upright and inverted faces are denoted by dashed and solid lines, respectively.

**Figure 3:**
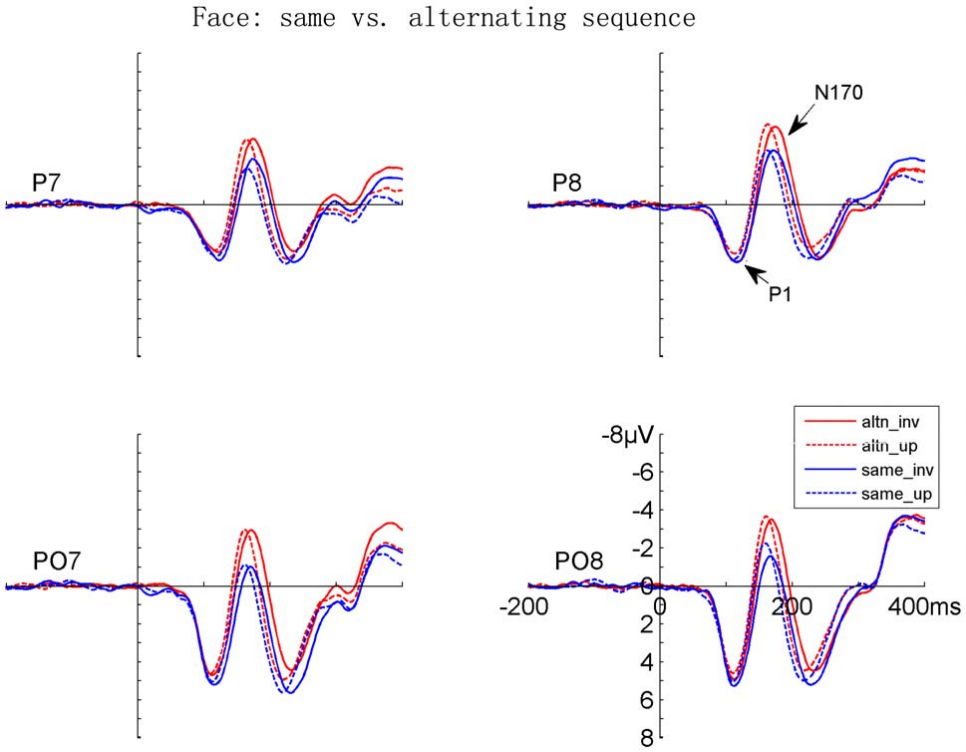
The grand-averaged ERPs elicited by upright and inverted faces under same and alternating orientation conditions. Data from the occipito-temporal (PO7/PO8) and temporal (P7/P8) sites are shown. The ERPs under same and alternating orientation conditions are denoted by blue and red lines, respectively. The ERPs for upright and inverted faces are denoted by dashed and solid lines, respectively. Note that the ERPs under the same orientation condition are the same as the ERPs under the short ISI conditions in Figure 2.

**Figure 4:**
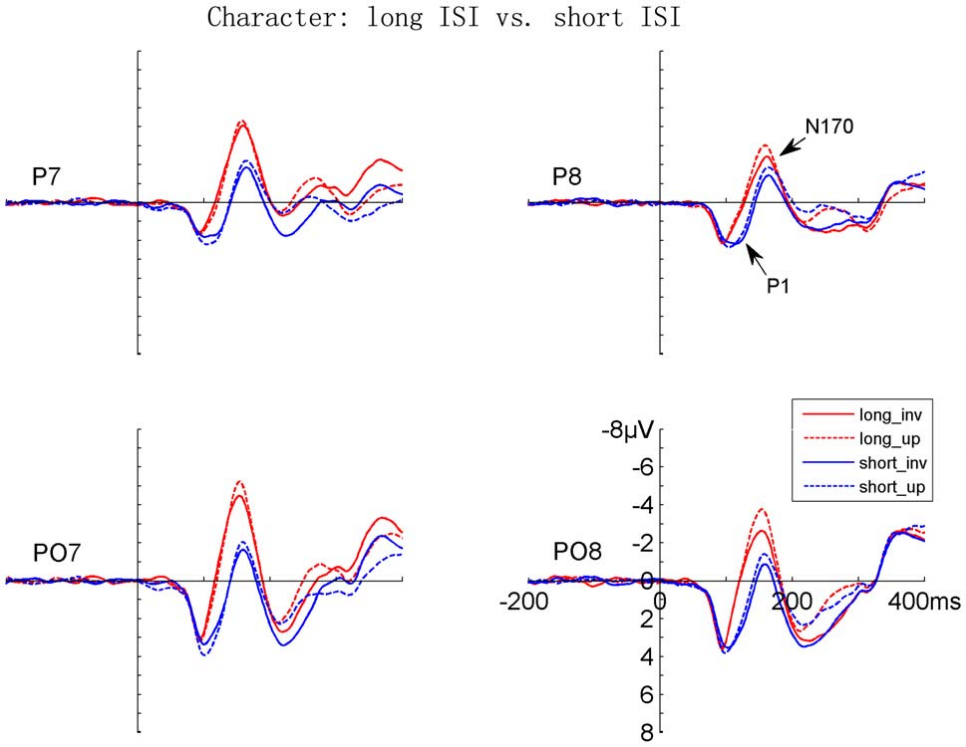
The grand-averaged ERPs elicited by upright and inverted Chinese characters under long and short ISI conditions. Data from the occipito-temporal (PO7/PO8) and temporal (P7/P8) sites are shown. The ERPs under long and short ISI conditions are denoted by red and blue lines, respectively. The ERPs for upright and inverted characters are denoted by dashed and solid lines, respectively.

**Figure 5:**
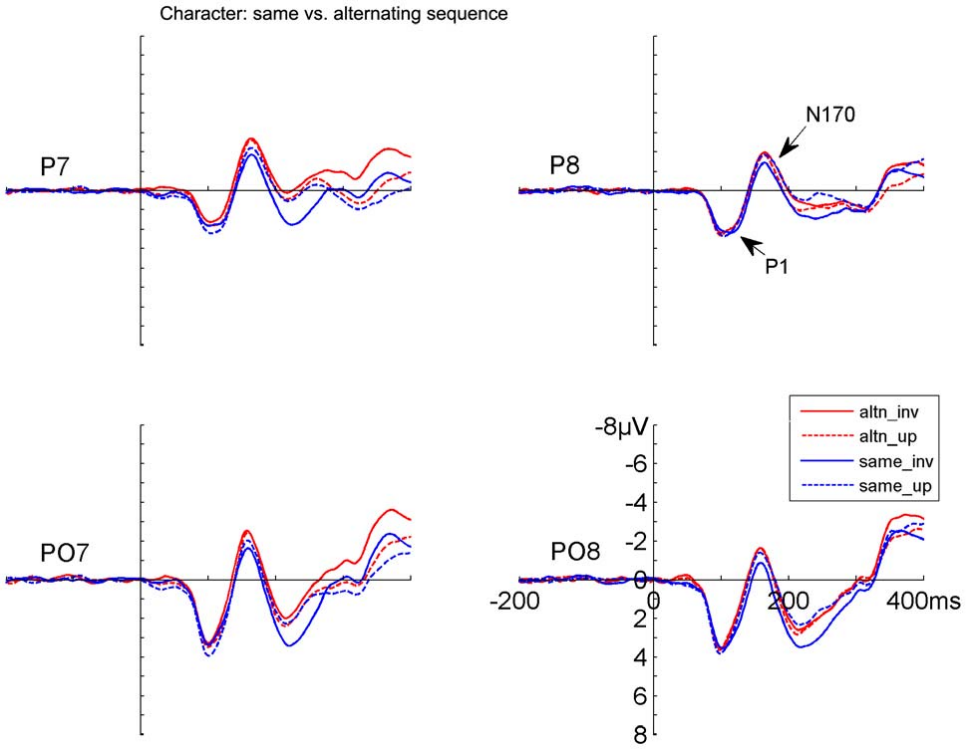
The grand-averaged ERPs elicited by upright and inverted Chinese characters under same and alternating orientation conditions. Data from the occipito-temporal (PO7/PO8) and temporal (P7/P8) sites are shown. The ERPs under same and alternating orientation conditions are denoted by blue and red lines, respectively. The ERPs for upright and inverted characters are denoted by dashed and solid lines, respectively. Note that the ERPs under the same orientation condition are the same as the ERPs under the short ISI conditions in Figure 4.

**Figure 6:**
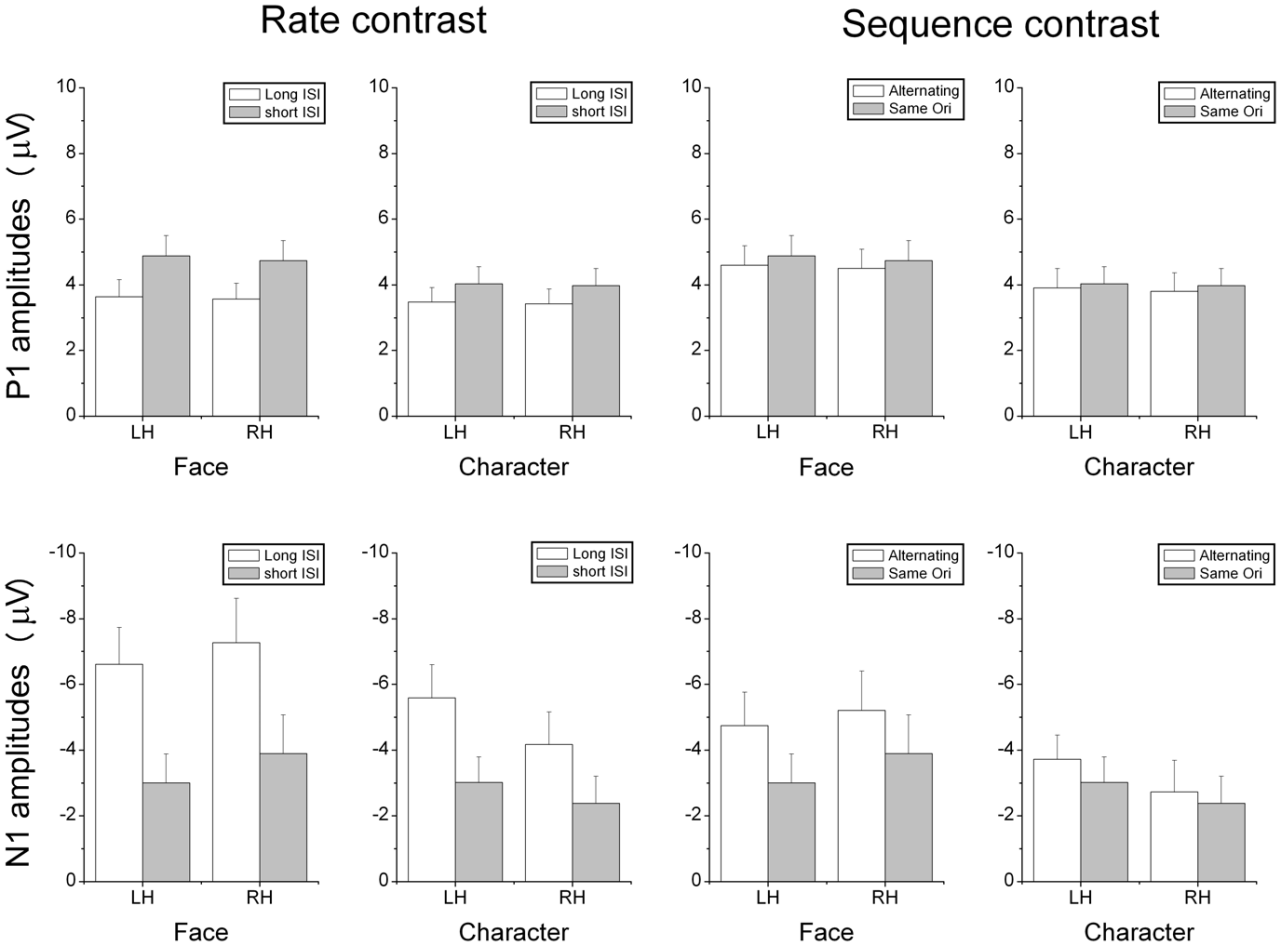
The mean amplitudes of the P1 and N1 components for faces and Chinese characters under rate contrast and sequence contrast. Data were presented for left hemisphere (LH, averaged across P7 and PO7 sites) and right hemisphere (RH, averaged across P8 and PO8 sites), respectively. Data were also averaged across orientation (upright vs. inverted).

**Figure 7:**
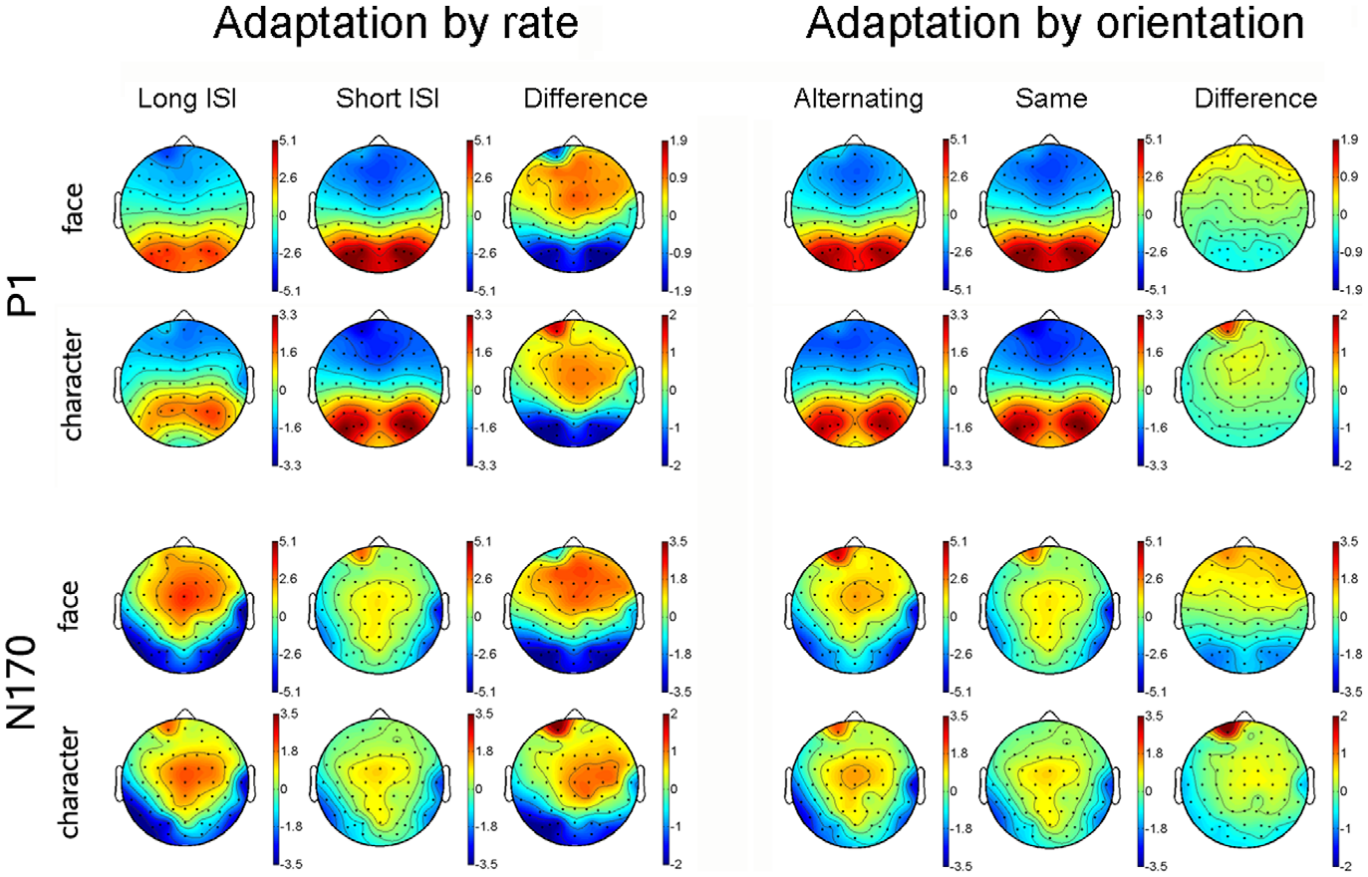
The scalp voltage maps of the P1 (averaged between 104–116 ms) and N170 (averaged between 164–176 ms) components elicited by faces and Chinese characters under conditions of long/short ISI and alternating/same orientation. Data were collapsed across upright and inverted stimuli. The voltage maps of adaptation-by-rate and adaptation-by-orientation effects were obtained by subtracting ERPs under the short ISI condition from that under the long ISI condition, and by subtracting the ERPs under the same orientation condition from that under the alternating condition, respectively.

Both faces and Chinese characters elicited clear P1 and N170 components, as shown in Figures 2, 3, 4, and 5. It is evident that the N170s between faces and Chinese characters showed clear differences (Figure 6). It is also evident that both the adaptation-by-rate and adaptation-by-orientation affected the scalp voltage distribution of the N170 for faces and Chinese characters (Figure 7).

### P1 Component

#### 1. Contrast between presentation rates (long vs. short ISI conditions)

Faces elicited a larger [F (1, 17) = 6.601, p<.020] and later [F (1, 17) = 19.096, p<.0005] P1 relative to characters. Both faces and characters showed an adaptation-by-rate effect, with a larger [F (1, 17) = 20.072, p<.0005] and later [F (1, 17) = 36.584, p<.0005] P1 under short relative to long ISI conditions (Figures 2, 4, and 6). This adaptation effect on the amplitude of P1 was more pronounced for faces relative to characters, as suggested by a significant Stimuli × ISI interaction [F (1, 17) = 5.461, p<.032], and was more pronounced at the occipito-temporal relative to temporal sites, as suggested by a significant Electrode × ISI interaction [F (1, 17) = 4.447, p<.050]. Separate analyses showed that the adaptation by rate effects on P1 amplitude was significant for both for faces and Chinese characters [F (1, 17) = 18.595, p<.0005, and F (1, 17) = 8.343, p<.010, respectively]. The amplitude of P1 was larger at the occipito-temporal relative to temporal sites [F (1, 17) = 31.772, p<.0005]. No inversion effect (i.e., upright vs. inverted stimuli) was found on the P1 component. The scalp voltage maps of the P1 components between the short and long ISI conditions are shown in Figure 7 (top panels on the left).

#### 2. Contrast between sequences (same vs. alternating orientation conditions)

Faces elicited a larger [F (1, 17) = 15.846, p<.001] and later [F (1, 17) = 25.610, p<.0005] P1 relative to characters. The adaptation-by-orientation effect was observed on the amplitude of P1, with a larger P1 under the same relative to alternating orientation conditions [F (1, 17) = 5.152, p<.037] (Figures 3, 5, and 6). Separate analyses showed that the main effect of Sequence on P1 amplitude was not significant, but the Sequence × Electrode interaction was significant [F (1, 17) = 9.152, p<.008] for faces, suggesting that the adaptation-by-sequence effect was larger at PO7/PO8 relative to P7/P8. For Chinese characters, the main effect of Sequence was not significant, but the Sequence × Electrode × Hemisphere interaction was significant [F (1, 17) = 4.832, p<.042]. This sequence effect on the amplitude of P1 was more pronounced at the occipito-temporal relative to temporal sites, as suggested by a significant Electrode × Sequence interaction [F (1, 17) = 6.531, p<.020]. The amplitude of P1 was larger at occipito-temporal relative to temporal sites [F (1, 17) = 29.590, p<.0005]. No inversion effect (i.e., upright vs. inverted stimuli) was found on the P1 component. The scalp voltage maps of the P1 components between the alternating and same orientation conditions are shown in Figure 7 (top panels on the right).

### N170 Component

#### 1. Contrast between rates (long vs. short ISI conditions)

Faces elicited a larger [F (1, 17) = 6.496, p<.021] and later [F (1, 17) = 5.105, p<.037] N170 relative to characters. This N170 enhancement for faces was more pronounced at the temporal relative to occipito-temporal sites, as suggested by a significant Stimuli × Electrode interaction [F (1, 17) = 5.739, p<.028]. The adaptation-by-rate effect was shown on both the amplitude and latency of N170, with a larger [F (1, 17) = 27.690, p<.0005] and earlier [F (1, 17) = 51.192, p<.0005] N170 under long relative to short ISI conditions (Figures 2, 4, and 6). The adaptation-by-rate effect on the amplitude of N170 was more pronounced for faces relative to characters, as suggested by a significant Stimuli × ISI interaction [F (1, 17) = 8.096, p<.011], and was more pronounced at occipito-temporal relative to temporal sites, as suggested by a significant Electrode × ISI interaction [F (1, 17) = 17.113, p<.001]. Separate analyses showed that the adaptation by rate effects on N170 amplitude was significant for both faces and characters, as suggested by significant ISI main effects [F (1, 17) = 65.261, p<.0005, and F (1, 17) = 40.765, p<.0005, respectively]. The N170 was more left-lateralized for characters relative to faces, as suggested by a significant Stimuli × Hemisphere interaction [F (1, 17) = 5.225, p<.035]. Inverted stimuli elicited a later N170 related to upright stimuli [F (1, 17) = 16.357, p<.001], and this inversion effect on the peak latency of N170 was more pronounced for faces relative to characters, as suggested by a significant Stimuli × Orientation interaction [F (1, 17) = 20.463, p<.0005]. No difference was found on the amplitude of N170 between the inverted and upright stimuli. The peak latency of N170 was earlier at the occipito-temporal relative to temporal sites [F (1, 17) = 22.957, p<.0005]. The scalp voltage maps of the N170 components between the short and long ISI conditions are shown in Figure 7 (bottom panels on the left).

#### 2. Contrast between sequences (same vs. alternating orientation conditions)

No significant main effect was found on the peak latency of N170 between faces and characters, but the Stimuli × Adaptation and the Stimuli × Adaptation × Electrode interactions were significant [F (1, 17) = 60.055, p<.0005; and F (1, 17) = 12.157, p<.003, respectively], suggesting that adaptation-by-orientation differentially affects the N170 latency between faces and characters (Figures 3, 5, and 6). The analyses of N170 amplitudes showed that faces elicited larger N170 relative to characters [F (1, 17) = 6.496, p<.021]. This enhanced N170 for faces was more pronounced at temporal relative to occipito-temporal sites, as suggested by a significant Electrode × Sequence interaction [F (1, 17) = 5.739, p<.028], and was more pronounced in the right relative to the left hemisphere, as suggested by a significant Hemisphere × Sequence interaction [F (1, 17) = 5.225, p<.035]. The adaptation-by-orientation effect on the amplitude of N170 was significant [F (1, 17) = 27.690, p<.0005], with a larger N170 for the alternating relative to the same orientation condition. This adaptation effect on the amplitude of N170 was more pronounced for faces relative to characters, as suggested by a significant Stimuli × Sequence interaction [F (1, 17) = 8.096, p<.011] and was more pronounced at occipito-temporal relative to temporal sites, as suggested by a significant Electrode × Sequence interaction [F (1, 17) = 17.113, p<.001]. Separate analyses showed that the adaptation by orientation effects on N170 amplitude was significant for both faces and characters, as suggested by significant Sequence main effects [F (1, 17) = 24.354, p<.0005, and F (1, 17) = 6.131, p<.024, respectively]. Inverted faces and characters elicited a later N170 [F (1, 17) = 27.751, p<.0005], and this inversion effect on the peak latency of N170 was more pronounced for faces relative to characters [F (1, 17) = 60.055, p<.0005]. The amplitude of N170 showed no difference between the upright and the inverted stimuli. The peak latency of N170 was earlier at occipito-temporal relative to temporal sites [F (1, 17) = 20.962, p<.0005]. The scalp voltage maps of the N170 components between the alternating and same orientation conditions are shown in Figure 7 (bottom panels on the right).

## Discussion

The neural correlates of face and Chinese character processing were investigated by comparing ERPs using two methods of neural adaptation, rate contrast (fast vs. slow presentation rate) and sequence contrast (same vs. alternating orientation). Three findings with the N170 ERP component pointed to a categorical difference between faces and Chinese characters: the amplitude of N170, its hemispheric distribution, and the latency delay of N170 for inverted stimuli. More importantly, different adaptation effects for faces and Chinese characters further pointed to a categorical difference between the two stimulus types. Physical differences between faces and Chinese characters cannot account for these results because they were balanced within each category for both the rate contrast and sequence contrast adaptation methods. As can be seen clearly in Figure 7, the N170 is larger for faces relative to characters, the distribution of occipito-temporal N170 is bilateral for faces and more left-lateralized for characters, and the adaptation effects on N170 elicited by rate and sequence contrasts are larger and more bilateral for faces relative to characters. Thus, the findings provide strong support for the domain-specific view of face processing. The present results extend previous findings of categorical differences between faces and English words [25], but do so in the context of better control over physical differences between faces and other categorical stimuli and in an adaptation paradigm that excludes the low-level property difference between two visual categories. The results also suggest that although both faces and Chinese characters require configural processing and afford high levels of visual expertise for literate Chinese readers, different neural mechanisms are involved in the perceptual processing of these two visual categories.

### Adaptation-by-rate (Rate Contrast) Effects on N170

Adaptation-by-rate effects were observed on the N170 component, with a larger and earlier N170 under the long relative to short ISI condition. To our knowledge, this is the first study showing that the N170 of Chinese characters can be modulated by adaptation contrasted between fast and slow presentation rates. Secondly, the N170 adaptation effect for faces is consistent with previous studies showing smaller N170 [18], [43] for repeated face stimuli. Finally, the N170 was later for short relative to the long ISI condition, suggesting that repetition suppression or adaptation, rather than repetition priming is likely to be involved in the present study. This is because a shorter N170 peak latency was reported for repetition priming in a previous study (Itier and Taylor, 2002).

It is possible to argue that the adaptation effect might be confounded with attentional effects on early negative brain activity. However, such an explanation seems unlikely, because attention generally enhances (rather than reduces) the N1 component [42] and thus the overlap of an enhanced N1 onto N170 would be an enhanced N170 for the fast presentation rate, which presumably required participants to pay more attention than the slow rate (e.g., see [44]). In contrast, a smaller N170 for the fast presentation rate was observed in the present study. In addition, participants responded equally fast to the face and character targets, suggesting that attention to these two stimuli might be comparable. Therefore, the different adaptation-by-rate effects on N170 between faces and characters suggest that perception and adaptation of faces and characters involves categorically different brain mechanisms.

### Adaptation-by-orientation (Sequence Contrast) Effects on N170

We also compared ERPs of faces and characters between the same and alternating orientation conditions, when both faces and characters were presented with short ISI. Unlike the rate contrast, this sequence contrast was not contaminated by attention or general arousal associated with different presentation rates. The only difference between the two sequences was whether all the stimuli had the same (either upright or inverted) or alternating orientation in different blocks. The categorical difference was found on the N170, with the faces showing a larger sequence effect relative to Chinese characters. Moreover, the inversion effect on the latency of N170, i.e., a later N170 for inverted relative to upright stimuli, was different between faces and Chinese characters, with a more pronounced N170 latency delay for inverted relative upright stimuli for faces. The implications of these results are as following. Firstly, the significant adaptation-by-orientation effect on N170 suggests that upright faces and characters are processed differently as compared with when they were inverted, otherwise no such effect should exist. Such an effect was obtained under the same presentation rate, and thus the general arousal or attention accounts are less likely as compared with the adaptation effects obtained by contrasting fast and slow presentation rates. Secondly, similar to the finding that inverted faces are processed differently relative to the upright faces [17], [45], the present results extend this finding to the processing of the inverted vs. upright Chinese characters. Thirdly, the different adaptation effects on the amplitude of N170 as a function of stimulus orientation suggest again that faces and characters involve categorically different brain mechanisms.

### Hemispheric Lateralization of N170

As shown in Figure 7, both faces and characters elicited an occipital-temporal N170, which was larger for faces relative to characters, especially over the right hemisphere. As can also be seen in Figure 7, the N170 was bilateral for faces and more left-lateralized for characters. In contrast to previous observations of a right-lateralized N170 for faces [6], [19], faces in the present study elicited a bilateral N170 without a hemispheric difference. There are several possible accounts for our finding of a bilateral N170 for faces. Firstly, faces have been reported to elicit bilateral or even left-lateralized N170 when presented among other faces [21], [46], [47]. Secondly, the lateralization of face processing seems to be affected by the duration of the stimulus, with more right-lateralized N170 under short duration (30 ms) condition but a more bilateral N170 under long duration (700 ms) conditions [48]. It is possible that the 200 ms duration in the present study is similar to the long duration, as N170 may have been fully developed after 200 ms. Finally, the color discrimination task in the present study might involve implicit processing, rather than explicit processing for faces. A recent study has shown that categorical (right dominant) perception of faces occurs only when faces are explicitly trained [49].

In contrast, the N170 of Chinese characters was more left-lateralized relative to the N170 of faces in the present study. Left-lateralized N170 has been observed for English words [19], [24], [48], Japanese Katakana or Kanji for native Japanese speakers [26], Korean [27], and Chinese characters and Roman [32]. It has also been reported that expertise affects the lateralization of the N170 elicited by syllabic and logographic Kanji of Japanese [26] and Chinese [32], with experts showing left-lateralized N170 and novices showing bilateral N170. Interestingly, however, bilateral N170 has been reported for Chinese characters when participants were Koreans who can read Chinese characters [27]. The left-lateralization of N170 of Chinese characters for native Chinese readers in the present study is consistent with those studies using expert participants (e.g., [26], [32]). Moreover, it is essential to control for low-level features when comparing ERPs between different stimuli, because the N170 of faces may be affected by inter-trial perceptual variance ([17], [50] for rebuttal, however). It has also been reported that N170s for faces and English words are differentially modulated by both stimulus size and resolution, and the lateralization of N170 for faces and English words is affected by psychophysical properties such as spatial frequency and presentation duration [48]. Relative to the contrast between faces and English words [19], [25], the present contrast between faces and Chinese characters controlled better for low-level properties, such as stimulus size and luminance.

### Inversion Effect of N170

While inverted faces usually elicit a larger and later N170 relative to upright faces [51], [52], the face inversion effect on the amplitude of N170 was absent in the present study. Nevertheless, a delayed N170, which is a more robust phenomenon than the amplitude enhancement of N170 for inverted relative to upright faces [6], [17], was observed in the present study.

Similar to faces, inverted Chinese characters also elicited a delayed N170 without amplitude change. This slightly differs from English words in that a larger and later N170 was elicited for inverted relative to upright English words [19]. However, similar to previous finding that the latency delay of N170 for inverted stimuli was more pronounced for faces relative to English words [19], the N170 latency delay for inverted stimuli was more pronounced for faces relative to Chinese characters in the present study. This different inversion effect on N170 latency suggests again that faces and Chinese characters are processed in a categorically different manner. Presumably, this is because stimulus inversion disrupts the perception of faces more than the perception of other non-face objects and non-face scripts, such as Chinese characters. However, a recent fMRI study has suggested that the processing of inverted Chinese characters may also recruit additional brain areas that might be related to object processing, relative to the processing of upright Chinese characters [53]. The present results provide little clue on the processing mechanisms between the inverted and upright Chinese characters and further research is needed to address the categorical difference between the inversion effects of faces and characters.

### Categorical Difference and Adaptation Effects on the P1 Component

It should be noted that the earliest categorical difference and adaptation effects were observed in the P1 component (approximately 120 ms after stimulus onset), before the N170 component, with the faces eliciting a larger and later P1 relative to Chinese characters. Similar difference on the amplitude of P1 was reported between faces and other objects in a previous study [5]. As discussed earlier, the direct contrast between different stimuli (such as faces and Chinese characters) can be confounded by difference in low-level stimulus properties, but the adaptation effects between faces and Chinese characters in the present study are not affected by physical difference. Moreover, the adaptation effects on P1 were observed under both rate contrast and sequence contrast conditions, with a larger and later P1 under the short relative to long ISI condition (rate contrast) and a larger P1 under the same relative to the alternating orientation condition (sequence contrast). One possible account for the rate effect on P1 was that attention co-varies with presentation rates, with more attention being paid to the stimuli in a fast relative to slow sequence. Firstly, this is supported the observations that attention enhances the amplitudes of P1 [42], [54]. Secondly, behavioral results are consistent with this attention account between the two presentation rates, because participants responded faster to stimuli under the short relative to the long ISI conditions. However, the adaptation-by-orientation effect on P1 was observed in the absence of RT difference, suggesting that attention cannot account for all the adaptation effects on P1. The role of low-level property, attention, and genuine categorical difference on the category and adaptation effects on P1 are still unclear.

### Limitations of the Present Study

It should also be mentioned that the present fast adaptation paradigm, as has been used in previous studies [41], is quite different from the traditional adaptation paradigm, in which the adaptor is often viewed for longer time (e.g., 180 s, [34]). Accordingly, the opposite pattern of adaptation effects on P1 and N1 component (e.g., enhanced P1 but diminished N1 for rate contrast) in the present study may prevent us from drawing strong conclusions on whether the N170 effect of rate contrast reflects “real” adaptation in a traditional way. Moreover, as can be seen clearly in Figure 7, the adaptation effects on both P1 and N1 point to a robust and interesting left-frontal difference between Chinese characters and faces. So far we are unable to provide interpretations for this categorical adaptation effect but can only speculate that this effect may be related to cognitive processing specific for Chinese characters, such as phonological and semantic processing [31], [55]. While these issues are of theoretical importance, the present study couldn’t address them in detail, given the scope and aims of the study and the limitation of data analysis. In addition, to further identify the categorical difference between faces and Chinese characters, future studies can be conducted using a design in which the neural mechanisms of these two visual categories are directly compared between the within-category adaptation (i.e., faces and characters are presented in separate blocks) and the between-category adaptation (i.e., faces and characters are alternated within a block) effects on P1 and N170.

In conclusion, domain-specificity of face processing was supported by the results of the present study, as indicated by different ERP response to faces relative to Chinese characters and by different ERP adaptation effects on N170 between them. A bilateral N170 was observed for faces, whereas a more left-lateralized N170 was observed for Chinese characters, suggesting hemispheric differences in the processing of these two stimulus categories. The adaptation effects on the amplitude of N170 were observed for faces and Chinese characters under both rate contrast (short vs. long ISI) and orientation contrast (same vs. alternating orientation) conditions. Importantly, this N170 adaptation effects also showed categorical differences between faces and characters, with better controlled low-level difference between these two visual categories. Moreover, faces and Chinese characters showed different neural adaptation effects as early as in the P1 time range (120 ms), although the underlying mechanisms are still unclear. We conclude that categorically different neural mechanisms are involved between faces and Chinese characters during perception and adaptation.
